# Defining Empowerment and Supporting Engagement Using Patient Views From the Citizen Health Information Portal: Qualitative Study

**DOI:** 10.2196/medinform.8828

**Published:** 2018-09-10

**Authors:** Tracie Risling, Juan Martinez, Jeremy Young, Nancy Thorp-Froslie

**Affiliations:** ^1^ College of Nursing University of Saskatchewan Saskatoon, SK Canada

**Keywords:** digital health, electronic health record, patient engagement, patient empowerment, patient portal

## Abstract

**Background:**

The increasing presence of technology in health care has created new opportunities for patient engagement and with this, an intensified exploration of patient empowerment within the digital health context. While the use of technology, such as patient portals, has been positively received, a clear linkage between digital health solutions, patient empowerment, and health outcomes remains elusive.

**Objective:**

The primary objective of this research was to explore the views of participants enrolled in an electronic health record portal access trial regarding the resultant influence of this technology on their feelings of patient empowerment.

**Methods:**

The exploration of patient empowerment within a digital health context was done with participants in a tethered patient portal trial using interpretive description. Interpretive description is a qualitative methodology developed to pragmatically address clinical health questions. Patient demographics, self-reported health status, and self-identified technology adaptation contributed to the assessment of empowerment in this qualitative approach.

**Results:**

This research produced a view of patient empowerment within the digital health context summarized in two overarching categories: (1) Being Heard and (2) Moving Forward. In each of these, two subcategories further delineate the aspects of empowerment, as viewed by these participants: Knowing More and Seeing What They See under Being Heard, and Owning Future Steps and Promoting Future Care under Moving Forward. This work also highlighted an ongoing interconnectedness between the concepts of patient empowerment, engagement, and activation and the need to further articulate the unique aspects of each of these.

**Conclusions:**

The results of this study contribute needed patient voice to the ongoing evolution of the concept of patient empowerment. In order to move toward more concrete and accurate measure of patient empowerment and engagement in digital health, there must be further consideration of what patients themselves identify as essential aspects of these complex concepts. This research has revealed relational and informational elements as two key areas of focus in the ongoing evolution of patient empowerment operationalization and measure.

## Introduction

Patient-centered care has long been promoted, debated, and pursued, yet substantial reform to established institutional approaches has proved challenging. The increasing presence of technology in health care delivery, coupled with consumer demand, is contributing to a remarkable shift in traditional practices and a renewed interest in patient empowerment as a means to advance this long-sought reform [1,2]. However, decades of debate and varied conceptual application have resulted in a lack of clarity in how to best operationalize or even consistently define patient empowerment [3-6]. Despite these challenges, recent reviews on patient empowerment reveal a global interest in this concept [2,3,5]. The World Health Organization (WHO) European Regional Office included empowerment and patient-centered practice as key elements in its Health 2020 report [7], a follow-up on previous WHO study on the effectiveness of empowerment to improve health [8]. In this earlier work, WHO identified empowerment as an essential public health strategy and also noted a scarcity of refined evaluative measures [8]. Many years later, this need for a comprehensive operational definition of patient empowerment and robust measures to evaluate the concept remains [2,3,5].

Publication on patient empowerment is increasingly present in digital health literature [9-12], with an emerging consideration that “the future of patient empowerment may lie in technological advancements and better access of patients to these technologies” [13]. As technology is promoted as a means to advance patient empowerment [9,10,14,15], the need to address contextual considerations in applying this concept to digital health has also been raised [12,16]. Patient empowerment has been examined in conjunction with technological apps such as electronic personal health records [17,18], patient Web portals [19], and electronic medical records [20]. Patient portals or “tethered” electronic personal health records are Web-based portals linked to electronic health records (EHRs) [14]. Studies on patient portals seem to have emerged as a focal point in this study, with connections made between portal use, patient empowerment, engagement, and activation and ultimately improved personal health outcomes [21-24].

Despite this promising beginning in the exploration of patient empowerment in digital health, a clear linkage among the use of digital health services, patient empowerment, and health outcomes remains elusive. Previous work has characterized the challenges of advancing a clear and comprehensive definition of patient empowerment in digital health app [3-5]. Such challenges emerge from the mixed or combined use of patient empowerment with terms such as patient engagement, enablement, activation, and even patient-centeredness, although calls for the distinct use and application of each of these conceptual entities have repeatedly been made [5,25,26]. A previously published scoping review, by these authors on patient empowerment measures, further highlighted the current discord in operationalizing patient empowerment [27]. The gaps and inconsistencies in the measures of empowerment are further exacerbated by negligible contributions of patient voice [3,27]. As such, incorporating patient perceptions into the development of patient empowerment measures has emerged as a critical need to disentangle the interconnectedness between patient empowerment, engagement, and activation, specifically with regards to the use of patient portals in digital health [3,27]. The primary objective of this qualitative study was to explore the influence of the patient portal use on patient views and perceptions of empowerment. Incorporating an underrepresented qualitative narrative with the current empowerment study maximizes opportunities for patient voice to direct patient-centered care while contributing to the needed delineation of empowerment aspects associated with patient portal use.

## Methods

### Study Aim

This study aimed to characterize participant experiences and views of empowerment related to the use of patient portals. This study was supported by a previous scoping review, by these authors, which explored current practices in the operationalization of patient empowerment in relation to the use of tethered patient portals [27].

### Recruitment, Appraisal of Health, and Technology Adaptation

Following the approval from the Research Ethics Board of the lead author’s institution, participant recruitment was conducted in collaboration with eHealth Saskatchewan, which, with the support of Canada Health Infoway, had recently deployed a limited launch of the Citizen Health Information Portal (CHIP). This tethered patient portal was provided to approximately 1000 provincial residents facilitating access to data contained in their EHR. The portal allowed patients to add medical history information and provided views of laboratory results, immunizations, prescriptions, and hospital discharge summaries, as well as an opportunity to set reminders for medications and appointments [28]. All CHIP participants received an email invitation to participate in the study. No geographical limits were imposed during recruitment, but participants did need to be English speakers. The recruitment process resulted in a purposive sample of 26 participants for the study.

Regarding the demographic data collection, participants were asked to self-identify their current health status. The primary means of accessing Web-based information was also assessed by self-report in this group. Finally, the research team used a modified adaptation of Rogers’ diffusion of innovations theory [29] to explore participant comfort in adopting and using new technologies. Rogers used the following 5 stages to identify how innovations are transmitted and taken up by members of a social system: (1) Innovators, these individuals are typically risk-takers and lead the way when it comes to new technologies; (2) Early Adopters, includes individuals who will be among the first to try new technologies; (3) Early Majority, represents individuals who take time to consider trying out something new before acquiring and using it; (4) Late Majority, includes the group of individuals that is somewhat cautious about trying new technologies and will tend to adopt the use of technology more slowly than the average; and (5) Questioners, representing the last group that will uptake a new technology with users that typically require proof that new technology is worth investing in [29].

### Data Collection and Analysis

Data collection was primarily achieved through semistructured interviews (Multimedia Appendix 1), and members of the research team also observed and collected data during a CHIP pilot participant focus group run by eHealth Saskatchewan. Once complete, the digital data files were transcribed, deidentified, and reviewed for accuracy, in preparation for analysis.

In this study, interpretive description (ID) was used, a qualitative methodology first detailed by Thorne et al [30]. ID was developed as a way of generating clinically relevant knowledge for health disciplines, and Thorne has since published, and recently updated, full text on this approach [31]. Initially, a detailed line-by-line coding of the data was undertaken within the transcript documents to provide an initial sense of the scope of the data. These early coding efforts revealed a wide diversity in the topic, enhanced by the line-by-line approach. The research team returned to the transcriptions to create a more focused dataset by collating all passages related to empowerment and engagement. In addition, the study team included any mention of either, or both, of these terms in the dataset based on the established interconnection of the concepts in the previously conducted scoping review [27].

The dataset was prepared in a split-page word document format, and the research team returned to the coding process. Moving away from the granular line-by-line approach, the data were instead examined, as Thorne recommended, through an “exploration of commonalities and differences among and between individual experiences” [32], reflective memoing was used during the analysis along with regular interchanges between research team members to share emerging interpretations and code categories. The ongoing dialogue supported the exploration of outlier data and allowed the team to come to consensus on how to integrate these findings. As the code categories began to coalesce and the interpretation emerged, the research team used the proposed descriptive categories and returned to the full transcript data set for a final review; this was done to ensure no other experiences or elements needed to be integrated into the analysis and final interpretation.

## Results

### Participant Demographics

**Figure 1:**
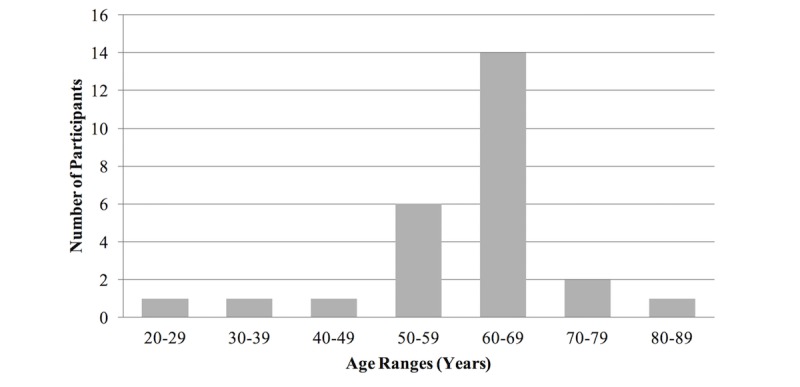
Participants’ age ranges.

All participants (N=26) resided in one Western Canadian province. The majority of participants were females (n=18), with fewer (n=8) male participants. Although the age of participants ranged 20-85 years, the predominant age category was 60-69 years (n=14), as shown in Figure 1.

**Figure 2:**
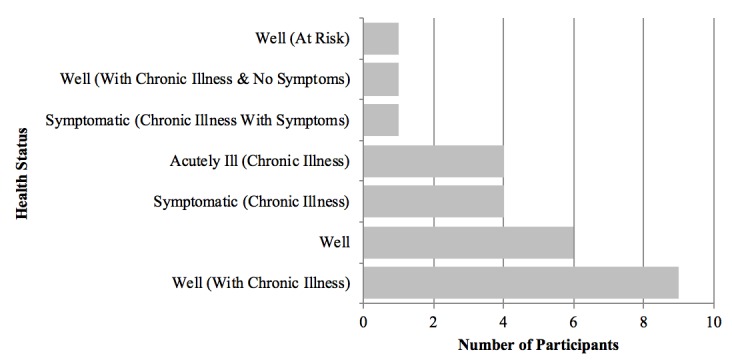
Self-identified health status of participants ranging from well to acutely ill with chronic illness(es).

**Figure 3:**
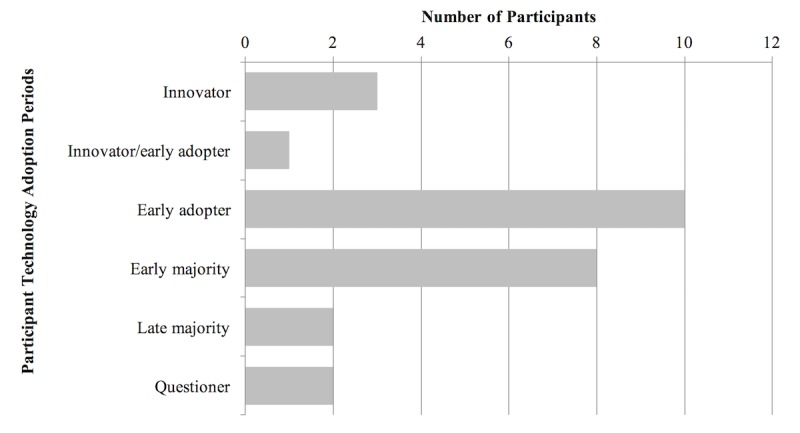
Technology adoption by participants.

As summarized in Figure 2, there was a diversity of self-reported health status, with the majority of participants rating their health within the well options. The way through which participants gained access to their health information on the portal varied, with most using a combination of personal computers, tablets, and mobile devices (n=15; data not shown). Figure 3 shows the range of self-identified technology adoption among the study participants, spanning the full scope of Rogers’ continuum.

### Interpretive Description Findings

#### Categories

**Figure 4:**
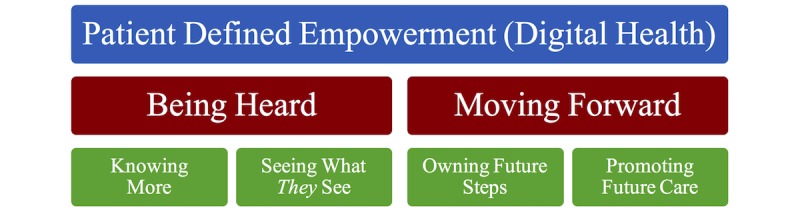
Interpretive categories identified in the qualitative study.

From the experiences shared by CHIP participants (N=26) in this qualitative exploration of empowerment and engagement, the following two overarching categories emerged: *Being Heard* and *Moving Forward*. For each of these, 2 subcategories were defined to delineate the aspects of empowerment and engagement further, as viewed by these participants: *Knowing More* and *Seeing What They See* (under *Being Heard*) and *Owning Future Steps* and *Promoting Future Care* (under *Moving Forward*). Figure 4 presents these categories, and each will be reviewed further.

ID is especially useful in considering the voice of the outlier or contrasting case [31]. In this way, ID is an opportunity to operationalize a patient-centered approach in the research app. In the case of this interpretive work on empowerment and engagement in relation to the patient portal experience, the research team encountered a participant whose view of empowerment was contrary to what had been commonly expressed. These data supported the emergence of the first overarching category in this interpretation, *Being Heard*.

#### Being Heard

Very early in the exploration of the participant transcripts, strong support emerged for empowerment, and many positive views about the concept were expressed. However, 1 participant did not seem to share this certainty when asked about the concept of patient empowerment, he responded:

I don’t know if I like that word. It is just encouraging to see that healthcare is going in a positive direction these days, and it just tells me and it makes me feel like I do have a say in my healthcare and how it’s administered to me and I want people to hear me the first time I present them with an issue, and that’s something I really appreciate.

Although the participant was unsure about the use of patient empowerment, there existed an essential element that arose from his statement about *being heard the first time* that resonated with the research team. A code category *Being Heard* was proposed, and the team returned to the data using the lens of this outlier view to determine whether other participants expressed similar sentiments. From this exploration, the overarching category of *Being Heard* was established. During the interpretative analysis, this category evolved to include 2 further subcategories or themes: *Knowing More* and *Seeing What They See*. Table 1 summarizes participant quotes supporting the development of this category and the included themes.

##### Knowing More

Pilot participants in the CHIP project could access their EHR, and a familiar sentiment about the availability of this new source of personal data was expressed by 1 study participant: “I guess I could go back to the old adage where you hear people say information is power.” In general, there was a significant response to the value of having direct access to personal health care data, especially as laboratory and medication information was updated in near real-time. This knowledge was often connected to the sense of empowerment, as demonstrated in the quoted excerpts in Table 1. Furthermore, the access to information was a foundational element of empowerment for these participants and a primary driver for their ongoing interactions with the portal. Moreover, *Knowing More* was a crucial foundation to support the pursuit of *Being Heard*.

##### Seeing What They See

The second element of *Being Heard* was a focus on what could be seen. Moving beyond the experience of simply *Knowing More*, *Seeing What They See* reflected not only the information but also an empowered sense of shared access to timely health care data with providers themselves. This access supported a variety of patient initiatives, as detailed in a few patient excerpts highlighting this theme in Table 1. Although some mixed feelings were reported regarding having access to test results, as well as differing opinion on whether this would increase or decrease physician workload, the overall sense of equality in being provided the same type of information that their own providers received was remarkable for study participants. There were recommendations that additional information, or suggestions, from providers to support self-care or behavioral changes, based on the data available in the portal, be incorporated in future offerings.

Together, *Knowing More* and *Seeing What They See* represent the empowered view of *Being Heard*. Overwhelmingly, study participants shared views that demonstrated that they were looking for opportunities to not only be further engaged in their own health care but also feel as though they were valued and knowledgeable team members in the decision making and delivery of this care. This sense of empowerment in future direction was represented in the second category of *Moving Forward*.

#### Moving Forward

This second overarching categorization comprised 2 subcategories or themes, *Owning Future Steps* and *Promoting Future Care*; these summarize the views of participants first in relation to their own self-care and second regarding their hopes and expectations for future technological advancements to support their ongoing involvement and engagement in their health care. Table 1 details participant quotes supporting this category and resultant themes.

##### Owning Future Steps

Although many participants in this study reported a history of substantial involvement in directing their own health, there was a profound sense of the significance of this portal technology in cementing the role of patients in the care relationship, especially in relation to decision making and engaging in self-care behaviors, as depicted in several quotes in Table 1. Where *Being Heard* was about taking in information and feeling empowered in the patient-provider relationship, *Moving Forward,* and in particular, *Owning Future Steps*, represented the resulting action when patients worked from an empowered position. Finally, the significance of the portal access in the lives of these participants, and its influence on their views of an evolving digital health care landscape, was summed in *Promoting Future Care.*

##### Promoting Future Care

Participants in this study provided a powerful contrasting view of a commonly held perception about the willingness of older patients to engage in technological health care solutions. *Promoting Future Care* not only highlights the desire of these participants to have continued access to the citizen portal but also for other technologies that could provide a more connected and supported health care future; details of this can be seen in the participant quotes for this theme in Table 1.

**Table 1:** Participant quotations supporting the themes in the category *Being Heard*.

Category and theme		Quotations
**Being Heard**		
	Knowing More	“I mean when I say empower, it empowers me, I would say it just gives me the confidence to know whether I’m asking the right questions or I have asked the right questions.” “It (the CHIP information) makes me a little bit more empowered to help make those decisions.” “Empowerment, so you can understand and know what’s going on.” “To be empowered means to be aware of what is happening with your health.” “I can look up and see that information, make decisions, have less repetitive questions with my doctor and focus on the things that I really need to know.” “It’s helping me assume responsibility and to be knowledgeable and I think to just be better prepared when I go see the doctor so I can ask meaningful questions.” “You’re not anxious about it and, you know, if you’re worried about something, you have a resource now that kind of tells you where to go with it or helps you determine where to go with it.” “I think it provides me with some peace of mind, because like I said, instead of hoping and being pretty sure it’s all fine, you can look, and you can know.”
	Seeing What They See	“If I can see what they are seeing maybe in a little bit in advance, then I can be better prepared when I go to my doctor’s office; like have my list of questions ready.” “I go back and look at my numbers, try to make some changes right there. So it gives me more up-to-date information and not having to wait to get in to see the doctor if there was something that’s off. If it’s something that I’d try to control myself like the blood sugar levels or A_1C_ then that’s something that I can take into my own consideration.” “CHIP has literally been a lifesaver for me, because I’ve been able to get my results before I even see the doctors, I’m able to formulate the questions that I need to ask the doctor and get far more engaged in my health care.” “To be empowered means to be aware of what is happening with your health, and test results can help that. Information from doctors and health care people can help that, and that way you can make the changes and adjustments that you may need to be well.”
**Moving Forward**		
	Owning Future Steps	“When I become engaged in my healthcare and utilizing the resources I can access, I try to garner enough knowledge and understanding to feel empowered to take the next step.” “It has become very apparent the importance of informed self-care, not taking the doctors and the health professionals out of the equation, but to be able to take some of that information and look at what I can do as an individual to help myself.” “I need to be able to manage that, because I’m the one that’s doing it every day.” “I can see that it’s vital, the way they we’re moving, that we have that self-empowerment that comes from using this information, having access and using this information ourselves.” “Well, I think something like that will give you more empowerment because it’ll give you a track to follow, it should give you something to follow, and I think that there’s even something else that could be done with that, in terms of empowering your healthcare.”
	Promoting Future Care	“I think if you want people engaged in the health system, you need to make the health system accessible, and I think this is certainly one way to do it.” “I think this type of technology will also empower physicians to be able to give patients better care, or more timely care.” “So I think that when you talk about technology and what it means, I think it’s just endless. There’s so much and I think it really has a future that will allow for a whole different kind of coordination and communication.” “CHIP is wonderful. I want very much for it to continue and I would encourage over time for more information to be shared on it, I mean things like perhaps radiologist reports and those kinds of things.” “I think it’s a great idea, I really do. We live in a technology age, we’re in an online age. People want information at their fingertips, they do a lot of this kind of stuff from home or from their smartphone or whatever. I think this is an idea whose time is here.” “I personally think it’s amazing, obviously, it has its limitations, but the only challenge will be presenting it in a way that people with different demographics and all types of background will accept it and be on board.” “I have mixed feelings about the increasing presence [of technology]. I love the access, I like the idea that if I needed care in a hurry people would have access to my information. I think that the more that happens the better, but the trade-off of that is the risk of somebody going in and looking at your information to use it to do you harm.” “Well, I’m probably as ambivalent as a lot of people are. Worrying a bit about security of information, enjoying having the access to it but still kind of okay, is it secure? How secure is it?” “Technology is, I mean I see it as a double-edged sword. It’s lifesaving and also costly as heck. I’m sure this whole CHIP thing doesn’t come for free but as a service I think it’s very—however much it costs— I’m hoping it’s not too much that they don’t find that it’s not worthwhile, because I think that it is (worthwhile) if it’s affordable.”

**Participant quotations identifying challenges or limitations with patient portal use.**

- “The amount of information that’s there, you know it’s going to be overwhelming for some people.”
- “I don’t really think it changed anything, because I knew enough to ask.”
- “I sometimes don’t think it’s always necessary for a doctor to give you that information unless there’s an interpretation required.”
- “I don’t know that I want to be more engaged. I don’t know enough about it so I just sort of trust that my doctor’s doing what’s best.”
- “The patient engagement is for people who want it, not everybody is going to want it.”

Though enthusiastic about the integration of technology into their health care futures, the study participants also expressed practical views regarding the potential impact of this shift in access and delivery. Concerns about privacy, cost, and the importance of continuing to focus on people in care were expressed. As has been noted, ID promoted the inclusion of outlier views in the interpretative process. Textbox 1 summarizes some key contrasting participants’ views about the portal technology and engagement in this type of access.

The use of ID serves as a very effective reminder for researchers, practitioners, and digital solution providers that no single initiative is going to meet every need regarding patient engagement and empowerment. The ongoing exploration of views, like those represented in Textbox 1, is key to recognizing not only potential research limitations but also those in the larger digital health milieu. Overall, a strong positivity associated with the expanding role of technology in health care, especially for services like patient portals, which allowed for improved patient access to their health care data.

## Discussion

### Principal Findings

Although there has been a considerable amount of academic exploration and publication on patient empowerment, a recent analysis revealed that a clear definition of the concept is still lacking [3,5,6]. In addition, there has been little consideration about the influence digital health may have on key attributes of patient empowerment. Overall, these findings support an existing challenge with the operationalization of this concept for use in digital health research. Following a previously published scoping review on patient empowerment measure in digital health [27], this qualitative study was conducted to contribute the needed patient voice to the ongoing discourse on empowerment in digital health and provide insight for the development of future measures.

Elements of the aspects of empowerment identified in this study through *Being Heard* and *Moving Forward*, and their respective subcategories, can be noted in previously articulated views of empowerment summarized by Bravo et al [3] in their recent analysis of patient empowerment: first, in this widely used definition by Funnell et al [33] from the early 1990s, “We have defined patient empowerment as the discovery and development of one’s inherent capacity to be responsible for one’s own life. People are empowered when they have sufficient knowledge to make rational decisions, sufficient control and resources to implement their decisions, and sufficient experience to evaluate the effectiveness of their decisions” and second in the work of Lau [34] “Patient empowerment begins with information and education and includes seeking out information about one’s own illness or condition, and actively participating in treatment decisions.”

The alignment of this qualitative interpretation with previous work on conceptualizing patient empowerment is a positive finding; however, it is important to note further complexities that have been proposed through the views of these participants, particularly those that relate to the digital health context. Findings highlighted in *Promoting Future Care* speak to a desire by patients for the continued ability to be able to access their data electronically, as well as their hopes for the interchange of data in the future to support new means of communicating with providers and ultimately more efficient care. However, there are also unresolved concerns represented in these participant views, regarding the privacy and security of information, technology costs, and for some, uncertainty about whether or not the data are actually wanted.

A direct connection among digital health, patient empowerment, and particular health outcomes have been difficult to clearly demonstrate, with conflicting reports on the success of interventions summarized in several previous systematic reviews [35-41]. This challenge is reflected in this study as well in contrasting participant views on particular aspects of this digital health solution. However, despite concerns regarding costs or potential security risks associated with digital data, participants in this study still overwhelmingly identified portal access as an empowering force in the management of their health; this is clearly reflected in *Knowing More*. The impact of having digital access to critical health data appeared to be transformative for participants and supported enhanced preparation for practitioner visits, as a well as an improved sense of well-being or “peace of mind.”

CHIP participants identified a positive association between the offered digital health solution and their sense of patient empowerment, which may prompt further reflection on how a lack of patient participation in empowerment conceptualization and measure, as identified by Bravo et al [3], may have contributed to the inconclusiveness of some digital health empowerment study [35-41]. An enhanced understanding of empowerment incorporating patient views is clearly needed to produce a more robust conceptualization. Only when this foundational work is complete should attempts to advance the evaluation and measurement of this concept move ahead. With these considerations, future measures should more accurately demonstrate the effectiveness of digital health tools in supporting patient empowerment. This study has provided a piece of this foundation by using patient voice to provide a set of conceptual elements from which the work of creating a fulsome operationalization of patient empowerment within digital health can begin.

The results of this study can be used to reflect on the aspects of patient portals that may have the most “empowering effects.” In particular, the combined participant views presented in *Knowing More*, *Seeing What They See*, and *Owning Future Steps* lend support to features such as the timely release of diagnostic results and the ability of patients to contribute data into their EHR. Evidence suggests that interventions, such as patient portals, can improve self-efficacy by giving patients tools for enhanced self-management, which, in turn, may contribute to a heightened sense of patient empowerment [9,18]. This study further highlights the empowering sense of equality that results when patients feel they have access to the same data as their health care providers, when they are *Seeing What They See*. Several patients noted a change in the dynamics of their provider relationships and identified the timely delivery of information as a critical element for their ongoing engagement in their health management. Furthermore, the ability to securely exchange information through a portal was acknowledged as a priority for more active patient collaboration and provides support to ongoing advocacy efforts for increasingly open data adoption in digital health solutions.

### Limitations

This study is situated in the context in which it was conducted and represents the views of pilot CHIP participants. As such, it is not generalizable or representative of the experiences of all CHIP users or of other patients who are engaged in tethered patient portal use. This interpretation of patient empowerment within a digital health context, focused on the portal use, may however resonate with other patients, practitioners, and service providers. Certainly, it has added much needed patient voice to the ongoing academic pursuit of the comprehensive conceptualization of patient empowerment.

### Conclusions

Providing patients electronic access to timely personal health information is a crucial step in supporting a new era of collaborative care. In this study, the use of a tethered patient portal (CHIP) introduced participants to the benefits of technology in supporting positive health outcomes and their own empowerment. CHIP participants shared their views of patient empowerment situated within the context of this digital health experience. This qualitative study produced a more in-depth patient-centric view of patient empowerment, including additional considerations relevant to the operationalization of this concept within a digital health context. This study supports the development of a patient empowerment measure to more effectively capture the influence of digital health initiatives on this vital outcome. The development of this instrumentation is the primary objective of ongoing research by members of this study team.

## Appendix

Multimedia Appendix 1 Semistructured interview guide.

## Abbreviations

CHIP: Citizen Health Information Portal
EHR: Electronic health record
ID: interpretive description
WHO: World Health Organization
