# Supplementary material for: Defining Empowerment and Supporting Engagement Using Patient Views From the Citizen Health Information Portal: Qualitative Study
Source: JMIR Med Inform. 2018 Sep 10;6(3):e43. doi: 10.2196/medinform.8828 (PMC6231721; doi:10.2196/medinform.8828)
Supplement: Multimedia Appendix 1 [file medinform_v6i3e43_app1.pdf]

Opening Questions:

What comes to mind when you think about your healthcare experience in SK?

Do you feel engaged in your health journey?

What actions do you take, or how specifically do you stay engaged?

What role has your personal health data (lab results, medical documentation, etc.) played for you in terms of your health in the past?

Tell me about your past experiences accessing your personal health care data?

What significance does the accessibility of your health information have on your feelings of engagement or participation, if any?

Additional Questions:

What does it mean to you to be empowered in your healthcare?

Tell me about your recent experiences accessing your healthcare data electronically through the Citizen Health Portal?

What are your thoughts about the role of technology in your healthcare future?

How do you feel about the increasing presence of technology in healthcare?

Closing Questions:

Is there anything you would like to tell me that we have not talked about?

Is there anything you would like to ask me?

This is a Multimedia Appendix to a full manuscript published in JMIR Medical Informatics. For full copyright and citation information see http://dx.doi.org/10.2196/medinform.8828
